# Smoking Cessation Strategies for Different Types of Cigarette Users Using a Digital Peer–Supported App and Nicotine Aids: Prospective Study

**DOI:** 10.2196/75876

**Published:** 2025-08-05

**Authors:** Shota Yoshihara, Kayoko Takahashi, Chiaki Uemura, Shin Murakami, Daichi Harada, Ying Jiang, Hiroshi Yamato

**Affiliations:** 1 Department of Rehabilitation Sciences, Graduate School of Medical Sciences, Kitasato University, 1-15-1, Kitasato, Sagamihara, Kanagawa, 252-0373, Japan, 81 0427889694; 2 A10 Lab Inc, Tokyo, Japan; 3 Department of Occupational Therapy, School of Allied Health Science, Kitasato University, Sagamihara, Kanagawa, Japan; 4 Department of Health Development, Institute of Industrial Ecological Sciences, University of Occupational and Environmental Health, Japan, Kitakyushu, Fukuoka, Japan

**Keywords:** smoking, heated tobacco product, digital therapeutics, peer support, digital peer support app, mobile health

## Abstract

**Background:**

Smoking cessation plans under Japan’s national health insurance system are hindered by low completion and success rates. A small-group intervention combining nicotine replacement therapy with digital peer support demonstrated improved smoking cessation success outcomes. However, the extent to which the type of tobacco products used affects the program’s efficacy remains unclear.

**Objective:**

This study aimed to evaluate the differences in smoking cessation success rates among cigarette-only smokers, heated tobacco product (HTP)–only users, and individuals who use both (dual smokers), following a group-based intervention combining nicotine replacement therapy and a digital peer–supported app.

**Methods:**

A prospective study involved smokers from Japanese workplaces who owned smartphones. Participants received free nicotine replacement therapy (either patches or gum) and access to a digital peer support app. This app facilitated anonymous group chats (up to 5 participants) to encourage interactions and smoking cessation efforts by sharing activity reports, including photos and comments. Participants were classified into 3 groups: cigarette-only smokers, HTP-only users, and dual smokers. Logistic regression analysis was conducted to compare cessation success rates, with cigarette-only smokers being the reference group (odds ratios [ORs] and 95% CIs).

**Results:**

A total of 435 participants were included in the final analysis, comprising 163 cigarette-only smokers (37.5%), 218 HTP-only users (50.1%), and 54 dual smokers (12.4%). The participants had a mean age of 46.6 (SD 10.1) years, with a predominant male representation (416/435, 95.6%) and a significant proportion (296/435, 68.1%) having more than 20 years of smoking history. The smoking cessation success rate was significantly higher among HTP-only users than among cigarette-only smokers (63.3% vs 52.8%; adjusted OR 1.84, 95% CI 1.57‐2.16). Conversely, dual smokers exhibited a nonsignificantly lower success rate than cigarette-only smokers (48.1% vs 52.8%; adjusted OR 0.96, 95% CI 0.79‐1.16).

**Conclusions:**

A group-based smoking cessation program using a digital peer support app yielded higher success rates among HTP-only users than among cigarette-only smokers. However, no significant differences were found in dual smokers. These findings highlight the importance of considering tobacco product type in workplace cessation programs.

## Introduction

Heated tobacco products (HTPs) are available in more than 40 countries [1], with Japan representing the largest global market [2]. In Japan, HTPs have become the second-most prevalent tobacco product [3]. According to the 2023 National Health and Nutrition Survey [3], 38.5% of male smokers and 42.3% of female smokers reported using HTPs. Among smokers, the distribution of tobacco product use was as follows: for men, 60.5% used only combustible cigarettes, 29.2% consumed only HTPs, and 9.2% were dual users; for women, the corresponding figures were 56.2%, 35.3%, and 7%, respectively [3].

HTPs heat tobacco rather than burn it, producing an aerosol (vapor) instead of smoke [4]. They have been marketed as alternatives to combustible cigarettes [5]. However, chemical analyses reveal that HTPs, despite emitting lower concentrations of combustion-related toxicants, still contain harmful substances similar to those found in cigarette smoke, particularly when additives like glycerol are considered [6]. Additionally, the proportion of Japanese smokers intending to quit has declined since 2014, coinciding with the rise of HTP use [3]. These findings suggest that, like combustible cigarettes, promoting cessation of HTP users remains a public health concern [7,8].

In Japan, a standard outpatient smoking cessation program has been made available for smokers with nicotine dependence [9]. Since 2020, Japan’s national health insurance has also covered this program for HTP users. This program includes a 12-week nicotine replacement therapy using nicotine patches, delivered over 5 sessions, along with counseling provided by physicians either in person or via telemedicine [10,11]. Nevertheless, the completion rate of this smoking cessation program remains low [9].

Systematic reviews confirm the efficacy of digital-based therapies, such as smartphone apps, in aiding smoking cessation [12,13]. Additionally, recent studies have examined differences in smoking cessation success rates by cigarette type (combustible cigarettes vs HTPs) using app-based interventions, suggesting that HTP users achieve higher smoking cessation success rates than those using combustible cigarettes [14,15]. However, these findings are based on individually focused apps, with research on group-type apps that may incorporate peer support remaining scarce.

A meta-analysis [16] indicated that group-type interventions incorporating peers enhance smoking cessation success rates. Moreover, a digital peer–supported app, known as a group-type app, allows individuals to encourage one another in their efforts to enhance healthy behaviors [17-20]. Although digital peer–supported apps may represent a potentially effective tool for smoking cessation, no studies have explored whether their effectiveness differs according to the type of tobacco product used.

This study aimed to examine the differences in smoking cessation success rates among cigarette-only smokers, HTP-only users, and dual users who participated in a group-based smoking cessation program that integrated nicotine replacement therapy with a digital peer–supported app.

## Methods

### Study Design

This prospective study aimed to determine whether smoking cessation success rates vary by type of tobacco product, using a digital peer–supported app combined with nicotine patches or gums. The study targeted current smokers used at 4 companies between June and September 2023. Participants were categorized into 3 groups: cigarette-only smokers, HTP-only users, and dual smokers. The primary outcome was the smoking cessation success rate, compared among HTP-only users, dual smokers, and cigarette-only smokers, the latter serving as the reference group.

### Participants

Current smokers enrolled in the smoking cessation program and using the digital peer–supported app were assessed. To be eligible, individuals were required to own either an iOS or Android smartphone and be enrolled in their company’s corporate health insurance program. Participants were defined as smokers if they reported smoking at least one cigarette per day, including novel tobacco products such as HTPs and e-cigarettes.

### Smoking Cessation Program

**Figure 1.**
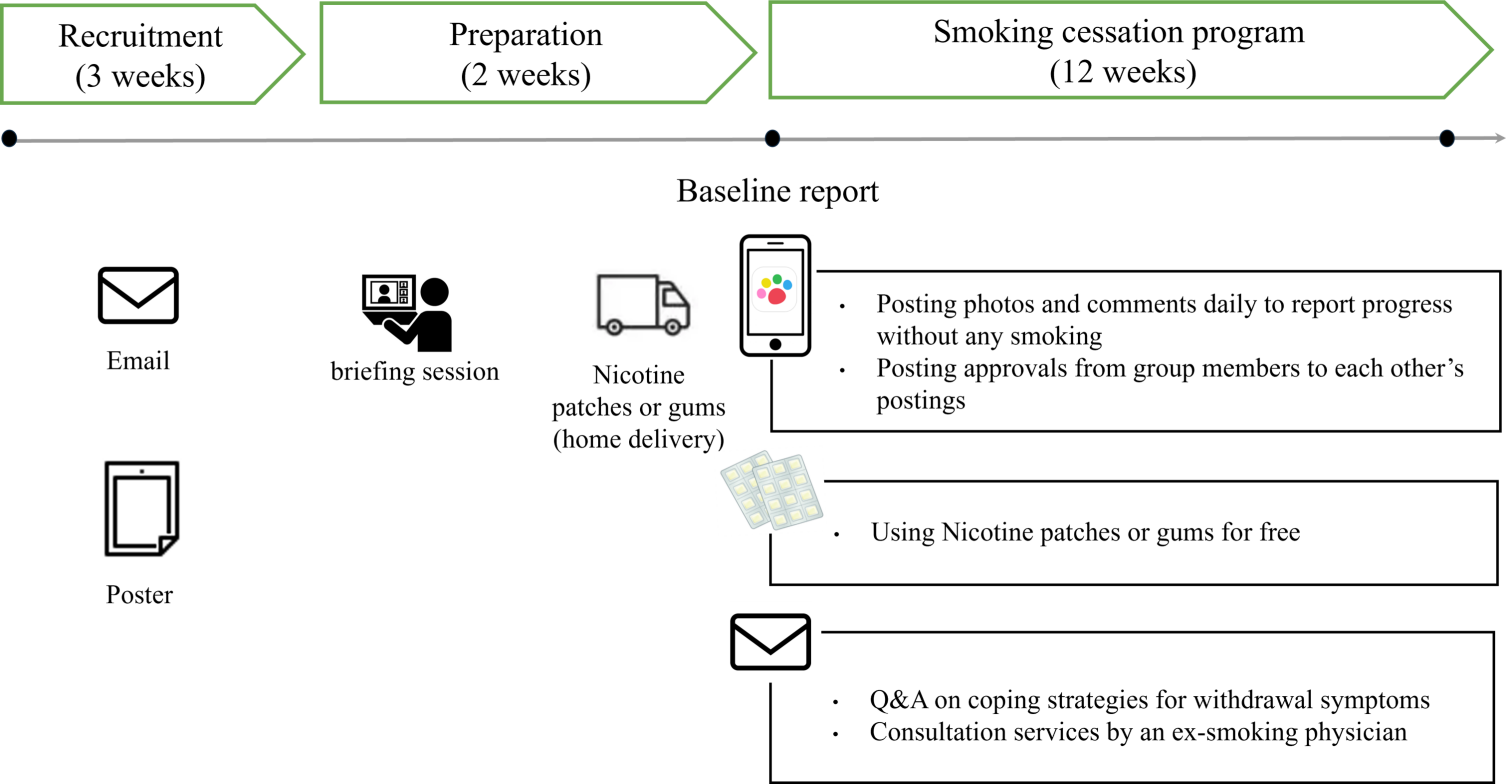
Timeline of study procedures. Q&A: question and answer.

Our smoking cessation program lasted 12 weeks and was conducted remotely for each company (Figure 1). Recruitment occurred over approximately 3 weeks, beginning about 2 weeks before the program, through 2 methods: individual outreach and workplace promotion. Participants were invited to join primarily via email from their companies and union officials associated with their health insurance societies. Additionally, posters and other promotional materials were displayed on company bulletin boards to raise awareness. Applicants joined this smoking cessation program by independently submitting their applications using the provided form (eg, Google Form).

During the preprogram phase, a real-time web-based videoconferencing briefing session was held to explain the program structure. This included educational content led by a physician who quit smoking. The session covered topics such as the health effects of smoking, the benefits of quitting, coping with withdrawal, creating a smoke-free environment, and adopting alternative behaviors. It also introduced the digital peer–supported app and demonstrated the proper use of nicotine patches or gums. Participants were provided with either nicotine gum or nicotine patches, based on their work environment and personal preference. Nicotine patches were included as an alternative for participants whose workplaces prohibited gum chewing during working hours (eg, automobile, food, and cosmetics factories). For those unable to attend the live session, the same content was made available as an e-learning course.

Following the web-based videoconferencing briefing session, participants received complimentary nicotine patches or gums, which are available over the counter, and began their smoking cessation efforts using the digital peer–supported app. Additional support included a “Q&A on Coping Strategies for Withdrawal Symptoms” provided through a dedicated email inquiry service and consultation services offered by an ex-smoking physician specializing in smoking cessation.

### Digital Peer-Supported App

In this study, we used “Minchalle,” a commercially available digital peer–supported app accessible on both Android and iPhone [17-20]. This app was originally developed to help users build desirable habits, such as exercising, dieting, and learning English conversation, and has been downloaded more than 1.6 million times. For this study, the app was adapted for smoking cessation support.

**Figure 2.**
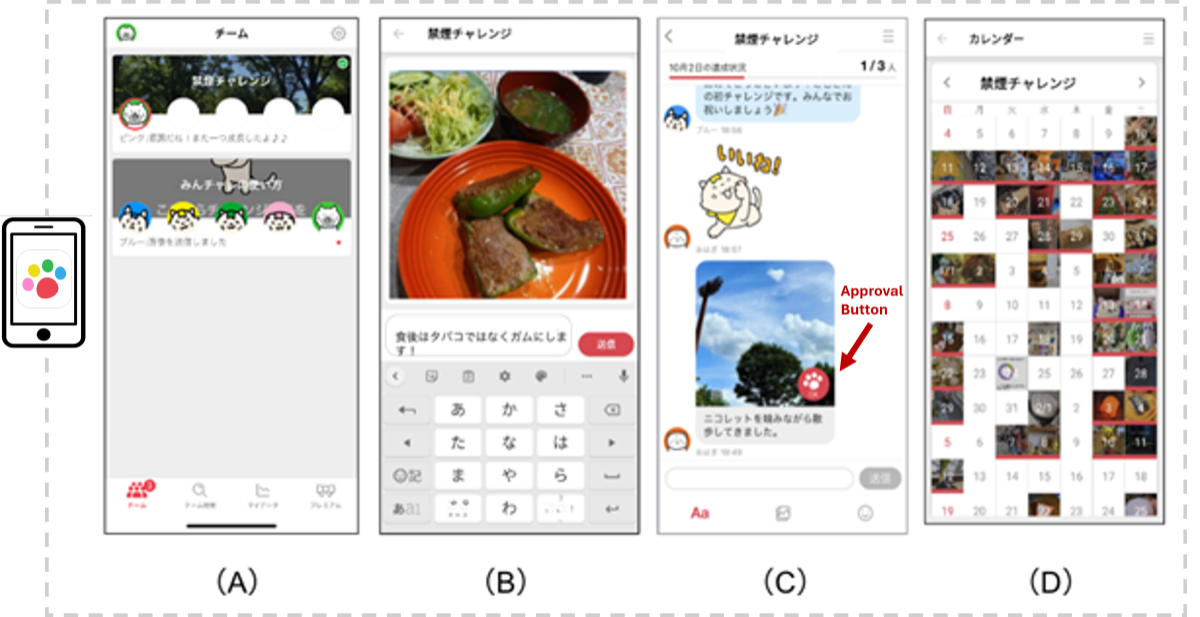
Examples of app screens. (**A**) Select a group of 5 members. (**B**) Upload a photo of the day with a comment related to abstinence. (**C**) Respond to posts by group members and press the approval buttons. (**D**) Look back at the records of abstinence through photos.

Participants were automatically assigned to teams of up to 5 individuals based on their registration order. The app facilitated group chats among team members sharing a common goal (in this case, smoking cessation) and enabled anonymous interaction through the posting of messages and photos related to smoking abstinence (Figure 2). To minimize potential social desirability bias, particularly given that participants were coworkers, all app interactions were designed to be anonymous. This anonymity encouraged candid communication and reduced the potential influence of workplace dynamics or hierarchical relationships on participants’ engagement and reporting behavior.

The app’s main objectives included (1) posting photos and comments daily to report progress without any smoking, (2) posting approvals from group members to each other’s postings, (3) receiving feedback on the team’s collective performance toward its goals, and (4) automatic removal from the group if a participant exceeded a specified inactivity period of 15 consecutive days. Participants could post comments or photos multiple times a day and interact with others as they wished, although participation was not mandatory. Participants could use the app for free. Examples of the user interface of the digital peer support app can be found in Multimedia Appendix 1.

### Measurements

#### Grouping by Types of Cigarettes Used

At baseline, cigarette smoking status was assessed using the question: “Which type of cigarette do you currently use?” Participants could select from multiple options: (1) combustible cigarettes, (2) HTPs (eg, IQOS, Ploom, Glo, PULZE, and WEECKE), or (3) e-cigarettes. We did not consider e-cigarette use because the prevalence of e-cigarettes was low in Japan [21-23], and e-cigarettes on the Japanese market do not contain nicotine [24].

Based on responses, participants were categorized into 3 groups: cigarette-only smokers, HTP-only users, and dual smokers.

#### Baseline Characteristics

Baseline data were collected via a web-based survey and included age (continuous), sex (male, female), and smoking-related characteristics: duration of smoking, number of cigarettes smoked per day, history of smoking cessation aids, purpose for smoking cessation, motivation level for smoking cessation, and type of cigarettes smoked.

The duration of smoking (years) was assessed using the question, “How many years have you been smoking?” Participants were asked to select one of the following options: 0‐2, 3‐4, 5‐9, 10‐19, 20‐29, or 30 years or more. The duration of smoking was measured without distinguishing between types of tobacco products. As HTPs have been available for less than 10 years, HTP-only users with a smoking duration exceeding 10 years were assumed to have previously used combustible cigarettes but are currently using only HTPs. Cigarette consumption was evaluated using the question, “How many cigarettes do you currently smoke per day?” Participants were asked to choose from the following options: 1‐10, 11‐20, 21‐30, or 31 or more. The history of smoking cessation aids was recorded as yes or no. The purpose of smoking cessation included my health, savings for money, family health, and others. Motivation levels for smoking cessation at baseline were rated on a scale from 1 to 10.

Composite variables were created based on participants’ responses to identify concurrent e-cigarette use within each smoking category: (1) cigarette-only smokers who also used e-cigarettes, (2) HTP-only users who also used e-cigarettes, and (3) dual users who also used e-cigarettes. These variables were used in descriptive analysis and included as covariates in the regression model to adjust for the potential confounding effects of e-cigarette use.

Participants were grouped based on their attendance during the preprogram phase into 3 groups: those who attended the live online session, those who completed the e-learning course, and those who did not attend either (classified as absent).

#### Log Data of the Digital Peer–Supported App

Usage data from the digital peer–supported app were extracted from the app’s database during the program. This included the posting frequency, defined as the total number of user-generated posts recorded during the smoking cessation program period. The total number of posts was included in 4 types of photos: “photo-attached comments,” “photos,” “comments,” and “stamps.”

#### Outcome (Smoking Cessation Success Rates)

Smoking cessation success rates were determined based on participants’ self-reported abstinence from smoking for at least 4 consecutive weeks. At the end of the 12-week program, participants submitted the date they last smoked via a web-based form. Participants were considered to have successfully quit smoking if the number of days between their self-reported “last smoking date” and the final report date was at least 4 weeks. Those who reported complete abstinence during this period, without consuming a single cigarette, were classified as “quitters.” Participants who did not submit a report were categorized as “non-quitters.”

### Statistical Analysis

A logistic regression analysis was conducted to compare the smoking cessation success rates of the cigarette-only smokers (as a reference) with those of the HTP-only users and dual smokers. Several steps were taken for statistical adjustment. Model 1 was adjusted for age (continuous) and sex (male or female). Model 2 included additional adjustments for the duration of smoking (0‐2, 3‐4, 5‐9, 10‐19, 20‐29, and 30 years or more) and the number of cigarettes smoked per day (1‐10, 11‐20, 21‐30, and 31 or more). Since previous studies in Japan suggested that participants may perceive e-cigarettes as a smoking cessation aid, we considered the impact of e-cigarette use [25]. Therefore, Model 3 included an additional adjustment for e-cigarette use (yes or no). In all models, worksites were incorporated as clusters (4 companies).

Statistical analyses were conducted using Stata (version 17.0 ; Stata Corp). Statistical significance was set at 0.05 (2-tailed). The odds ratios (ORs) and 95% CIs were used to quantify the strength of associations between variables.

### Ethical Considerations

This study was approved by the Research Ethics Committee of the Institutional Review Board of the School of Allied Health Sciences at Kitasato University (approval number 2024‐008). At the time of program enrollment, participants were also informed that data obtained through their involvement in the program might be used for research purposes in an anonymized form, with participation being contingent upon their agreement to these terms. An opt-out method was used to obtain informed consent, with study details publicly disclosed online. Participants could withdraw at any time. No financial compensation was offered. All data were anonymized to ensure privacy.

## Results

**Figure 3.**
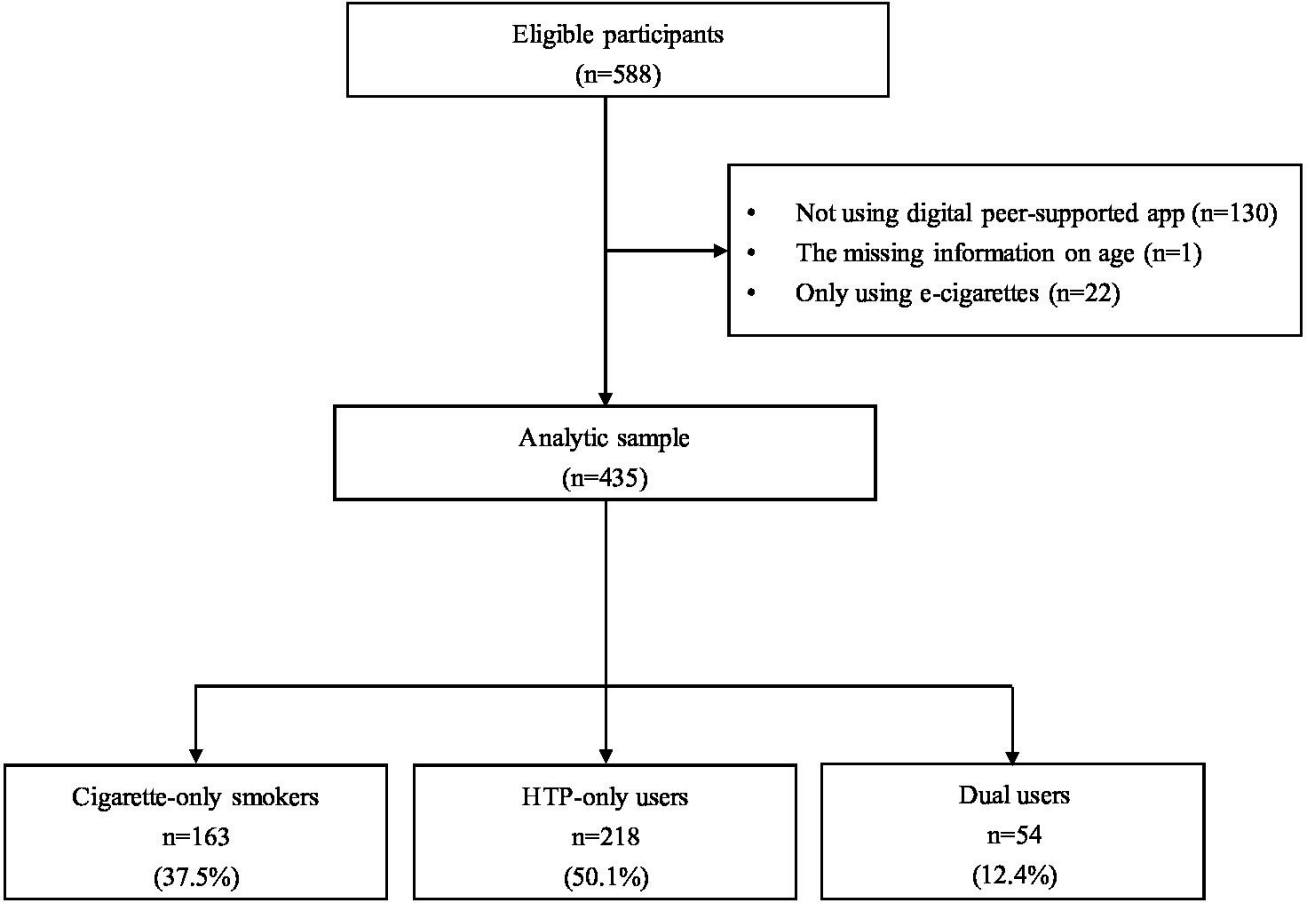
Flow diagram of study participants. HTP: heated tobacco products.

The full eligibility assessment and enrollment process in the study is illustrated in Figure 3. A total of 588 participants enrolled in the program, with 130 (23%) not using the digital peer support app and 1 participant missing age data. The final analysis included 435 participants. Of these, 163 (37.5%) were classified as cigarette-only smokers, 218 participants (50.1%) as HTP-only users, and 54 participants (12.4%) as dual smokers.

Table 1 summarizes the baseline characteristics of the participants. The mean age was 46.6 (SD 10.1) years, and 95.6% (416/435) of participants identified as male. Additionally, 68.1% (289/435) had used tobacco for 20 years or more. HTP-only users had a shorter smoking history and a lower proportion of previously used smoking cessation aids than cigarette-only smokers.

**Table 1.** Characteristics of cigarette-only smokers, heated tobacco product (HTP)–only users, and dual smokers.

Characteristics		Tobacco product types				*P* value
		Total (N=435)	Cigarette-only smokers (n=163)	HTP-only users (n=218)	Dual smokers (n=54)	
Age (years), mean (SD)		46.6 (10.1)	49.1 (9.4)	46.2 (9.1)	41.2 (13)	<.001
Sex, n (%)						.96
Male		416 (95.6)	156 (95.7)	208 (95.4)	52 (96.3)	
Female		19 (4.4)	7 (4.3)	10 (4.6)	2 (3.7)	
Duration of smoking (years), n (%)						<.001
0‐2		11 (2.5)	4 (2.5)	2 (0.9)	5 (9.3)	
3‐4		11 (2.5)	1 (0.6)	5 (2.3)	5 (9.3)	
5‐9		30 (6.9)	6 (3.7)	17 (7.8)	7 (13)	
10‐29		87 (20)	27 (16.6)	48 (22)	12 (22.2)	
20‐29		153 (35.2)	59 (36.2)	81 (37.2)	13 (24.1)	
30+		143 (32.9)	66 (40.5)	65 (29.8)	12 (22.2)	
Cigarettes/day, n (%)						.01
1‐10		105 (24.1)	54 (33.1)	39 (17.9)	12 (22.2)	
11‐20		231 (53.1)	83 (50.9)	117 (53.7)	31 (57.4)	
21‐30		87 (20)	22 (13.5)	56 (25.7)	9 (16.7)	
31+		12 (2.8)	4 (2.5)	6 (2.8)	2 (3.7)	
History of smoking cessation aids, n (%)		232 (53.3)	95 (58.3)	110 (50.5)	27 (50)	.28
Purpose of smoking cessation, n (%)						.12
My health		241 (55.4)	98 (60.1)	121 (55.5)	22 (40.7)	
Savings for money		122 (28)	40 (24.5)	65 (29.8)	17 (31.5)	
Family health		58 (13.3)	22 (13.5)	24 (11)	12 (22.2)	
Others		14 (3.2)	3 (1.8)	8 (3.7)	3 (5.6)	
Motivation for smoking cessation, mean (SD)		7.0 (2)	7.1 (2)	7 (2.1)	6.8 (2.2)	.51
Posting frequency in digital peer-support apps, mean (SD)		13.3 (27.7)	15 (30)	13 (27.3)	9.2 (21.3)	.40
E-cigarette use, n (%)		7 (1.6)	2 (1.2)	4 (1.8)	1 (1.9)	.89
Attendance rates for the preprogram, n (%)						.41
Live session		63 (14.5)	30 (18.4)	25 (11.5)	8 (14.8)	
e-Learning course		299 (68.7)	109 (66.9)	153 (70.2)	37 (68.5)	
Absent		73 (16.8)	24 (14.7)	40 (18.3)	9 (16.7)	

a Continuous variables were compared using the Kruskal-Wallis test for 3 groups. Categorical and binary variables were compared using Pearson chi-square test.

Table 2 shows the smoking cessation success rates achieved through the combined use of digital peer support with nicotine patches or gums, categorized by type of smoking product. For smoking cessation success rates, HTP-only users had higher rates than cigarette-only smokers, with an adjusted OR of 1.84 (95% CI 1.57‐2.16). Conversely, dual smokers had lower success rates than cigarette-only smokers (adjusted OR 0.96, 95% CI 0.79‐1.16).

**Table 2.** Odds ratios (ORs) comparing smoking cessation success rates among cigarette-only smokers, heated tobacco product (HTP)–only users, and dual smokers.

	Cigarette-only smokers (n=163)	HTP-only users (n=218)	Dual smokers (n=54)
Smoking cessation success, n (%)	86 (52.8)	138 (63.3)	26 (48.1)
Model 1^a^, OR (95% CI)^b^	1 (reference)	1.57 (1.44‐1.71)	0.87 (0.65‐1.16)
Model 2^c^, OR (95% CI)^b^	1 (reference)	1.84 (1.57‐2.16)	0.96 (0.79‐1.16)
Model 3^d^, OR (95% CI)^b^	1 (reference)	1.84 (1.57‐2.16)	0.96 (0.79‐1.16)

a Adjusted for age (in years, continuous) and sex (male or female).

b Logistic regression analysis.

c Adjusted for age (in years, continuous), sex (male or female), the duration of smoking (0‐2, 3‐4, 5‐9, 10‐19, 20‐29, and 30 years or more), and the number of cigarettes smoked per day (1‐10, 11‐20, 21‐30, and 31 or more).

d Adjusted for age (in years, continuous), sex (male or female), the duration of smoking (0‐2, 3‐4, 5‐9, 10‐19, 20‐29, and 30 years or more), the number of cigarettes smoked per day (1‐10, 11‐20, 21‐30, and 31 or more), and e-cigarettes use (yes or no).

## Discussion

### Principal Results

This study evaluated the effectiveness of a smoking cessation program that combined digital peer support with nicotine patches or gums among smokers using combustible cigarettes or HTPs. We found that HTP-only users had significantly higher success rates than cigarette-only smokers. This is the first study to assess the outcomes of combined digital peer support and nicotine patches or gums for HTP-only users compared with those for cigarette-only smokers.

### Comparison With Previous Studies

According to the 2023 National Health and Nutrition Survey [3], among male smokers aged 40‐49 years, 41.4% were HTP-only users, 50.9% were cigarette-only smokers, and 11.9% were dual users. Among female smokers in the same age group, 41.5% were HTP-only users, 43.9% were cigarette-only smokers, and 9.8% were dual users. Notably, among both men and women aged below 40 years of age, the proportion of HTP-only users exceeded that of cigarette-only smokers. These trends suggest that our study sample, composed primarily of working-age male adults, represents demographic groups exhibiting the highest prevalence of HTP use.

Our findings that HTP-only users achieved higher smoking cessation rates than cigarette-only smokers are consistent with those of previous studies comparing cessation outcomes between different tobacco product users in app-based interventions [14,15]. HTP-only users may have stronger motivation to quit and lower nicotine dependence, both of which are associated with greater cessation success [22,26,27]. However, our findings showed no significant differences in self-reported motivation to quit among cigarette-only smokers, HTP-only users, and dual users. Additionally, the cigarette-only group had a higher proportion of light smokers (ie, those smoking 1‐10 cigarettes per day: 54/163, 33.1%) compared to the HTP-only users (39/218, 17.9%) and dual smokers (12/54, 22.2%). These inconsistencies may be attributed to individual psychological and behavioral factors, such as personality traits, perceived harm, degree of team engagement, or the stage of behavioral change. These factors may affect app engagement and healthy behavior change [28].

Furthermore, our results are consistent with those of previous studies showing no significant differences in smoking cessation behavior between dual smokers and cigarette-only smokers [29]. One possible explanation is that dual smokers may already be actively attempting to quit smoking [26,27], although cigarette-only smokers may still be in the early stages of cessation. However, other studies have reported that dual users have lower cessation success rates than cigarette-only smokers [15,29,30], suggesting that not all dual users are necessarily in the early phase of behavior change.

To enhance the robustness of these findings, future studies should apply behavioral models, such as the stages of change framework [31], to better interpret observed discrepancies. It may also be beneficial to differentiate between subgroups of dual smokers, such as those who predominantly use HTPs, those who mainly smoke cigarettes, and those who primarily use HTPs but occasionally smoke cigarettes. Additionally, former smokers, particularly younger individuals, who used HTPs were at a higher risk of relapse within 1 year post cessation [32]. Similar patterns were observed among participants in the HTP-only group in this study. Therefore, future research should track individuals who successfully quit smoking to evaluate the risk and timing of potential relapse.

To address these complexities and improve outcomes, future app-based intervention designs could incorporate user segmentation to tailor support more effectively. This may involve accounting for individual characteristics such as motivation level and communication preferences, for example, whether a user prefers steady, independent progress or active peer interaction through messaging. Moreover, adaptive features that adjust content based on user engagement and behavioral stage (eg, the frequency of motivational messages, level of support, and use of community functions) might help improve adherence and perceived intervention relevance. Such personalized design approaches could help optimize digital peer support programs for diverse user needs and smoking patterns.

Importantly, this study does not endorse switching from cigarettes to HTPs as a cessation strategy. Despite limited scientific evidence supporting HTPs as an effective smoking cessation aid [33], approximately half of the current dual users in Japan reportedly use HTPs under the belief that they can facilitate smoking cessation [26]. Furthermore, previous studies have shown that exclusive HTP users are more likely than dual users to perceive HTPs as effective for smoking cessation [34].

### Limitations

This study has some limitations. First, the lack of a control group limits our ability to attribute the observed smoking cessation success solely to the app. However, in our proof-of-concept trial, the group using both smoking cessation aids and the digital peer–supported app showed a higher smoking cessation success rate (59.2% vs 38.7%) than the group using only smoking cessation aids. This study was designed based on these results [35]. Second, outcomes were self-reported, not biochemically verified, potentially affecting the accuracy of smoking cessation status. Although expert consensus indicates that biochemical verification is often impractical and unnecessary in similar studies [36], future research should consider incorporating such methods to enhance the validity of findings. Third, we did not account for potential confounding variables, including sociodemographic characteristics (eg, occupation, education, and income), smartphone usage patterns, adverse events, continued nicotine product use, or a history of smoking cessation [27]. Notably, age at smoking initiation, an essential factor associated with nicotine dependence and cessation outcomes, was not collected. Additionally, other potentially relevant factors such as alcohol or substance use, mental health conditions (eg, depression or anxiety), chronic diseases (eg, asthma or chronic obstructive pulmonary disease), nicotine dependence levels, and behavioral stages of change (eg, precontemplation, contemplation, preparation, and action) were not measured. Addressing these factors systemically in future studies would reduce bias and improve the interpretability of inter-group differences. Fourth, no formal sample size calculation was performed due to the lack of previous studies on this specific digital peer support smoking cessation program. Fifth, most HTP users (89%) were former cigarette smokers (“switchers”) with more than 20 years of smoking history. Although this reflects real-world usage patterns in Japan, motivations for switching to HTPs were not assessed. Understanding these may offer deeper insight into cessation behavior and app responsiveness in this subgroup. Sixth, activity-related indicators within the digital peer support app were limited to basic posting frequency. Future evaluations should incorporate detailed in-app behavior metrics (eg, message content, peer engagement quality, or feedback responsiveness) to better assess the mechanisms of behavioral change. Finally, the study sample consisted primarily of male employees from large Japanese companies, limiting the generalizability of findings to other populations, including women and nonworking individuals. 

### Conclusions

HTP-only users achieved higher success rates in group-based smoking cessation programs incorporating a digital peer support app than cigarette-only smokers. In contrast, no significant difference in cessation success was observed between dual users and cigarette-only users. These findings highlight the importance of considering the type of tobacco products used when designing and implementing smoking cessation support programs. These results underscore the importance of considering the type of tobacco product used when developing and implementing workplace smoking cessation interventions.

## Supplementary material

10.2196/75876 Multimedia Appendix 1 Examples of the user interface of the digital peer support app.

## Abbreviations

HTP: heated tobacco product
OR: odds ratio
